# Retraction Note: Downregulation of CDKL1 suppresses neuroblastoma cell proliferation, migration and invasion

**DOI:** 10.1186/s11658-025-00698-7

**Published:** 2025-02-07

**Authors:** Weiyi Li, Jing Cao, Jian Liu, Wenli Chu, Congqing Zhang, Shuiling Chen, Zefeng Kang

**Affiliations:** 1 Eye Hospital, China Academy of Chinese Medical Sciences, No 33 Lugu Road, Shijingshan District, Beijing, 100040 China; 2 Yinan Branch of Qilu Hospital of Shandong University, Linyi, Shandong China

**Retraction Note: Cellular & Molecular Biology Letters (2019) 24:19** 10.1186/s11658-019-0139-z

The Editor-in-Chief has retracted this article because of concerns regarding the figures presented in this work. These concerns call into question the article's overall scientific soundness. An investigation conducted after its publication discovered the following issues:

- The second *GAPDH* band in Fig. 1B appears to overlap with the *GADPH* band in Fig. 2E in [1];
- The third *CDKL1* band in Fig. 1B appears to overlap, when flipped and re-scaled, with the *Ftl1*, *Duodenum* band in Fig. 1A in [2];
- The micrograph shown in Panel *GFP*, *siCDKL1* of Fig. 2B appears to overlap with the micrograph shown in Panel *GFP*, *shZFR* of Fig. 2a in [3];
- The *GAPDH* band in Fig. 2D appears to overlap, when flipped and re-scaled, with the *GADPH*, *MG-63* band in Fig. 2C in [4];
- The assay shown in Panel *0h*, *NC* in Fig. 4A appears to overlap with the assay shown in Panel *0h*, *pcDNA* in Fig. 4A in [5].

The Editor-in-Chief therefore no longer has confidence in the integrity of the research presented in this article.

The authors have not replied to correspondence from the Publisher.
